# Septicaemia models using *Streptococcus pneumoniae* and *Listeria monocytogenes*: understanding the role of complement properdin

**DOI:** 10.1007/s00430-013-0324-z

**Published:** 2014-04-12

**Authors:** Aline Dupont, Fatima Mohamed, Nur’Ain Salehen, Sarah Glenn, Lorenza Francescut, Rozita Adib, Simon Byrne, Hannah Brewin, Irina Elliott, Luke Richards, Petya Dimitrova, Wilhelm Schwaeble, Nina Ivanovska, Aras Kadioglu, Lee R. Machado, Peter W. Andrew, Cordula Stover

**Affiliations:** 1 Department of Infection, Immunity and Inflammation, Maurice Shock Medical Sciences Building, University of Leicester, University Road, Leicester, LE1 9HN UK; 2 Department of Immunology, Institute of Microbiology, Bulgarian Academy of Sciences, Sofia, Bulgaria; 3 Department of Genetics, College of Medicine, Biological Sciences and Psychology, University of Leicester, Leicester, UK

**Keywords:** Complement, Mouse model, Bacterial infection, Dendritic cells, Macrophages, Fc receptor

## Abstract

*Streptococcus pneumoniae* and *Listeria monocytogenes*, pathogens which can cause severe infectious disease in human, were used to infect properdin-deficient and wildtype mice. The aim was to deduce a role for properdin, positive regulator of the alternative pathway of complement activation, by comparing and contrasting the immune response of the two genotypes in vivo. We show that properdin-deficient and wildtype mice mounted antipneumococcal serotype-specific IgM antibodies, which were protective. Properdin-deficient mice, however, had increased survival in the model of streptococcal pneumonia and sepsis. Low activity of the classical pathway of complement and modulation of FcγR2b expression appear to be pathogenically involved. In listeriosis, however, properdin-deficient mice had reduced survival and a dendritic cell population that was impaired in maturation and activity. In vitro analyses of splenocytes and bone marrow-derived myeloid cells support the view that the opposing outcomes of properdin-deficient and wildtype mice in these two infection models is likely to be due to a skewing of macrophage activity to an M2 phenotype in the properdin-deficient mice. The phenotypes observed thus appear to reflect the extent to which M2- or M1-polarised macrophages are involved in the immune responses to *S. pneumoniae* and *L. monocytogenes*. We conclude that properdin controls the strength of immune responses by affecting humoral as well as cellular phenotypes during acute bacterial infection and ensuing inflammation.

**Electronic supplementary material:**

The online version of this article (doi:10.1007/s00430-013-0324-z) contains supplementary material, which is available to authorized users.

## Introduction

Complement is an integral component of the antibacterial immune response. Its importance lies in its ability to recognise pathogen-associated molecular patterns, bound natural antibodies, charge clusters [1, 2] and hydrolysed C3. This recognition is translated into a sequential, tightly controlled activation of serine proteases using enzyme complexes, so-called convertases. C4b2b is the C3-converting enzyme of the classical and lectin pathways, whereas C3bBb cleaves more C3b from C3 as part of alternative pathway activation. The alternative pathway, in addition, amplifies the activity of complement. By the addition of nascent C3b to the enzyme complexes, their substrate specificity for C3 changes to target C5, generating C5a, a potent anaphylatoxin and C5b, the initiator of assembly in the target membrane of the membrane attack complex. While in the immediate response, crosstalk with coagulation and kininogen has been identified [3, 4], complement in the adaptive immune response co-determines the phenotypes of dendritic cells and T cells [5]. Properdin stabilises the C3 and C5 convertases, extending their enzymatic half-lives. Its relative importance in mouse models of sepsis and non-septic shock has been documented using genetically engineered properdin-deficient mice [6, 7]. Hereditary human properdin deficiency, possibly in epistasis with certain immunoglobulin allotypes, predisposes to meningococcal septicaemia [8].

While formation and insertion of the membrane attack complex are efficient against gram-negative organisms, encapsulated organisms activate complement in the non-immune host by binding of natural antibodies of the IgM-type [9]. In vitro, C3b deposition on the capsular pneumococcal serotype 2 strain D39 is mediated mainly by recognition of C1q to bound natural IgM, with some contribution by the alternative pathway of complement, consistent with the idea that the latter provides an amplification loop for increased C3b deposition [10]. In vivo, mice deficient in classical pathway activity (C1q deficient [P. Andrew, personal communication; [10]], C4 deficient [10]), in alternative pathway activity (Factor B deficient) [10], in lectin pathway activity (MASP-2 deficient, Ficolin A or Ficolin B deficient) [11, 12] or of C3 [13] was all significantly impaired in their survival of serotype 2 pneumococcal sepsis at 72 h compared to wildtype controls.

The alternative pathway is activated on the surface of *Listeria monocytogenes* [[14]; and own data]. Complement C5 is necessary to limit the number of viable *L. monocytogenes* retrieved from mouse organs after intravenous infection [15], most likely via intact chemotaxis and macrophage activation [16]. Complement receptors are known to be specifically involved in the uptake of *L. monocytogenes* by liver-resident Kupffer cells (CRIg) [17] and in the early phase of granuloma formation (CR3, which is CD11b/CD18 integrin) [18–20] in mouse models of listeriosis.

While natural antibodies provide protection against pneumococcal sepsis in mice [21], murine listeriosis is seen as a gold standard to assess cellular immunity [22]. This study investigates the role of properdin in gram-positive septicaemia by studying the phenotype of properdin-deficient mice and their wildtype controls in models of infection with *S. pneumoniae* and *L. monocytogenes*. Risk groups for these infections are as follows: for *S. pneumoniae* the very young and the elderly, and for *L. monocytogenes*, pregnant women and the immune compromised.

## Materials and methods

### Mice

Age-matched properdin-deficient and wildtype mice (C57Bl/6 background) were taken from the colony generated and maintained at the University of Leicester (8–12 weeks old) [6]. Experiments were performed in accordance with UK Home Office regulations and institutional ethical approval.

### Pneumococcal infection models

*Streptococcus pneumoniae* serotype 2, strain D39, was passaged in MF1 mice as standard procedure to maintain virulence. For infection, mice were anaesthetised with 2.5 % (v/v) fluothane (AstraZeneca, Macclesfield, UK) over oxygen (1.5–2 l/min), and pneumococcal suspension (1 × 10^6^ CFU in PBS, 50 μl) was administered intranasally (i.n. model). Intravenous infections were administered via the tail vein using the same dose (100 μl i.v. model). Dose concentration was confirmed by plating on blood agar. Mice were monitored for signs of illness throughout the 7 days of the experiment and culled before reaching the endpoint of severely lethargic. This timepoint was recorded as the survival time. Mice alive at 7 days post-infection were deemed to be survivors. Vaccination with Pneumovax was performed by single intraperitoneal (i.p.) injection as described [23].

### Determination of viable pneumococcal counts in lung homogenates and blood

Approximately 30 min, 6, 24 and 48 h after i.n. infection, mice were anaesthetised with 2.5 % (v/v) fluothane, and blood was collected by cardiac puncture. Mice were culled, and lungs were collected into 10 ml PBS and homogenised using an Ultra-Turrax T8 homogeniser (IKA-Werke, Germany). After i.v. infection, blood samples were collected from a tail vein at 24, 48 and 72 h. Viable counts from each homogenate and blood sample were determined by serial dilution using blood agar (Oxoid) containing 5 % (v/v) defibrinated horse blood and overnight incubation in an anaerobic jar at 37 °C. The colonies showed typical morphology for *S. pneumoniae* and α-hemolysis.

### Pulmonary C3 activation

Lung homogenates from control and infected mice were analysed by Western blot using HRP-conjugated goat antimouse C3 (ICN pharmaceuticals Inc.), and the density of bands corresponding to the C3 activation products was measured.

### IgM, anti-PPS2 IgM, C3 and C4c ELISA

Bloods were collected under terminal anaesthesia by cardiac puncture. Sera from infected and control mice (*S. pneumoniae* model) were stored in aliquots at –80 °C until required. Sandwich ELISAs were used to determine IgM (Bethyl Laboratories, Universal Biologicals Ltd., Cambridge UK) and total C3 levels (Immunology Consultants Laboratory Inc, Immune Systems Ltd., Paignton UK). To determine antipolysaccharide 2 IgM antibodies, plates were coated with purified serotype 2 pneumococcal polysaccharide (ATCC, Middlesex, UK) [24] and serum was added (1:300 dilution). Importantly, serum samples were pre-absorbed (37 °C, 1 h) with purified pneumococcal cell wall polysaccharide (CWPS Statens Serum Institute, Denmark; 5 µg/ml) to neutralise antibodies against cell wall polysaccharide before antibodies to PPS were detected using the antigen capture ELISA. C4c levels were determined using a previously described method [25].

### TNF-α levels

A bioassay was performed using actinomycin sensitised L929 indicator cells, and levels were expressed in relation to a TNF-α standard (Peprotech EC Ltd., London).

### Listeria infection model

*Listeria monocytogenes* serovar 1/2a, strain EGD-e (obtained from American Type Culture Collection) was passaged in C57Bl/6 mice. 1 × 10^6^ CFU in PBS were injected in a tail vein (100 μl). The number of viable, inoculated bacteria was determined by colony counts. The mice were monitored for signs of illness and culled before reaching a lethargic state. Mice alive at 96 h were considered to have survived the infection.

### Determination of numbers of viable counts of *L. monocytogenes* in liver

Mice were culled, and livers were homogenised in 10 ml PBS or dH_2_O as above. The numbers of CFU/g of liver tissue were determined by plating serial 10-fold dilutions of homogenates on BHI-agar plate (37 °C, 24 h). The colonies showed typical morphology (small, round colonies with a bluish-green sheen when viewed by obliquely transmitted light).

### IFNγ ELISA and nitric oxide measurement

Bloods were collected under terminal anaesthesia by cardiac puncture. Sera were prepared from infected and control mice (*L. monocytogenes* model) and stored at −80 °C. Murine IFN-γ ELISA Development Kit was used according to the manufacturer’s instructions (Peprotech). Nitric oxide production was measured using Griess Reagent kit (Promega, Wisconsin, USA).

### Differentiation of dendritic cells and macrophages from mouse bone marrows

Bone marrow cells were prepared using standard methodology by flushing tibias and femurs with PBS using a 0.45 mm syringe. The supernatant, after sedimentation of debris, was centrifuged, and erythrocytes were lysed using a hypotonic solution. After neutralisation, cells were seeded at 2 × 10^6^/ml in the presence of IL-4 and GM-CSF (each 10 ng/ml; Peprotech). After 7 days, non-adherent cells were separately collected from those cells, which became adherent under these conditions. Both populations were used separately for in vitro infection in antibiotic free complete culture medium (RPMI 1640, 10 % (v/v) FCS) with unpassaged, washed *L. monocytogenes* (at a multiplicity of infection, or MOI, of 0.2) in mid exponential growth phase, following the method described in a previous study [26].

### Cyclic amplification analyses

cDNA was transcribed from 1 μg total RNA prepared from cells or tissues using First Strand synthesis kit (Invitrogen). Gene-specific amplification using SensiMix SYBR kit (Bioline Reagents Ltd., London) was analysed with Rotor-Gene 6000 (Corbett Life Science) and expressed in relation to the housekeeping gene using the ΔΔCT method. Primer sequences for this were mouse FcgR2b: 5′-CTGAGGCTGAGAATACGATC-3′ and 5′-GTGGATCGATAGCAGAAGAG-3′; for mouse FcgR4: 5′-GTGACCCTCAGATGCCAAGGC-3′ and 5′-TGGATGGAGACCCTGGATCGC-3′, for mouse β-actin: 5′-GTGGGCCGCTCTAGGCACCAA-3′ and 5′-CTCTTTGATGTCACGCACGATTTC-3′, for mouse IL-17A 5′-GGCTGACCCCTAAGAAACC-3′ and 5′-CTGAAAATCAATAGCACGAAC-3′. Annealing temperatures were 60, 68, 55 and 53 °C, respectively.

Oligonucleotides used for standard amplification of murine transcripts for TLR2, CD11b and C3 (annealing temperature 55 °C) were 5′-GGCCAGGTTCCAGTTTTCAC-3′ and 5′-GGAACAACGAAGCATCTGGG-3′; 5′-GATGGTGTCGAGCTCTCTGCG-3′ and 5′-TTGTCTCAACTGTGATGGAGCA-3′; 5′-GAATACGTGCTGCCCAGTTT-3′ and 5′-TGAGTGACCACCAGCACTTT-3′, respectively.

### Flow cytometric analyses

To quantify the phagocytic uptake of *S. pneumoniae* D39 by splenic macrophages, extracellular fluorescence was quenched with 0.2 (w/v) % trypanblue prior to flow cytometry. To prepare the labelled bacteria, *S. pneumoniae* D39 were grown in broth (stationary, 37 °C), and 10^9^ bacteria/ml were resuspended in 0.1 mg/ml FITC (Sigma-Aldrich, 0.1 M NaHCO_3_ pH 9.0). After 60 min at room temperature, bacteria were spun and washed in PBS until the supernatant no longer was yellow. Splenic cell suspensions were prepared from a pool each of two properdin-deficient and wildtype mice, and adherent cells were incubated in the presence of genotype-matched serum with labelled *S. pneumoniae* for 1 h prior to analysis.

To characterise the dendritic cell phenotype after infection with heat-killed *L. monocytogenes* (MOI 0.2), surface expressions of CD11c and CD40, CD80, CD86 or MHCII, respectively (BD Biosciences Pharmingen, Oxford, UK), were analysed. Positive staining was compared with the relevant isotype controls (mouse IgG FITC or PE labelled, BD Biosciences Pharmingen). Compensation for different spectra was performed.

To investigate the level of FcγR2b expression, splenocytes were prepared from properdin-deficient and wildtype mice, infected overnight with heat-killed *L. monocytogenes* (MOI 20) and analysed with PE-labelled B-cell-specific rat antimouse CD45R/B220 and FITC-labelled rat antimouse CD16/CD32 (FcγIII/II receptor) (2.4G2) (BD Pharmingen).

Events were acquired using Becton–Dickinson FACS Scan and CellQuest pro software.

### Statistical analyses

Survival curves were compared using the log rank test. Other data were analysed using *t* tests. The difference in sequentially measured CFU in blood from infected mice was analysed by Mann–Whitney *U* test.

## Results

### Properdin is dispensable for protective immunity to *Streptococcus pneumoniae,* but significantly contributes to demise in acute pneumococcal pneumonia and sepsis

**Fig. 1:**
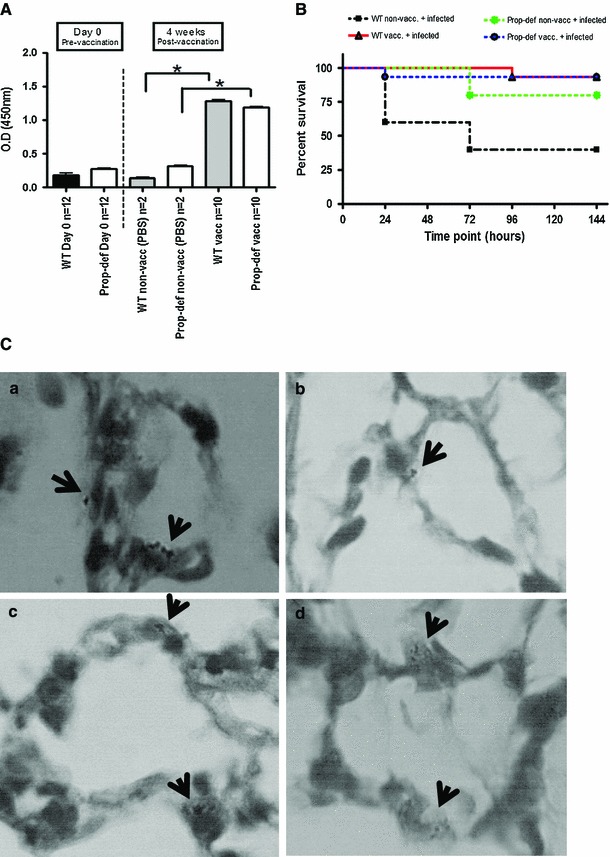
Properdin-deficient mice in pneumococcal vaccination and infection model. **a** Generation of anticapsular polysaccharide type 2 IgM antibodies after immunisation with Pneumovax: levels of IgM-specific antibodies responses to capsular polysaccharide type 2 after pre-absorption of sera with capsular wall polysaccharides (mean ± SD), pre- and post-vaccination. After vaccination with the polysaccharide vaccine, properdin-deficient and wildtype (WT) mice showed an increase in anticapsular type 2 IgM antibodies (*p* < 0.05) compared to non-vaccinated (PBS injected) mice. Importantly, these levels were reached at week 1, consistent with [28]. **b** Pneumococcal pneumonia and sepsis in untreated and immunised mice. Wildtype mice are protected during intranasal challenge with *S. pneumoniae* D39 (vaccination success), while properdin-deficient mice show greater survival independent of immunisation. The survival of infected properdin-deficient mice compared to infected wildtype mice is significantly better (*p* < 0.05). Viable counts were recovered from the bloodstreams of 2/5 infected, non-vaccinated mice of either genotype, whereas in the vaccinated and challenged groups, viable counts were detected in only one of the properdin-deficient mice. **c** Representative images of cocci present in alveoli at the endpoint of the experiment (day 7). Lung sections (*a* non-vaccinated and infected WT; *b* non-vaccinated and infected KO; *c* vaccinated and infected WT; *d* vaccinated and infected KO) were stained with Wright’s stain. ×100 oil immersion

**Fig. 2:**
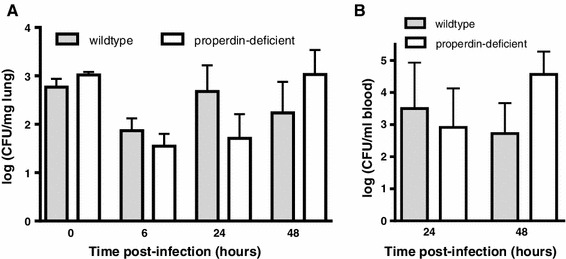
Viable pneumococci in lungs and blood of infected properdin-deficient and wildtype mice. **a** Wildtype mice limited bacterial numbers in lungs at 48 h compared to 24 h, while the numbers of viable bacteria were increased in properdin-deficient mice for these timepoints (*n* = 2 for t0, 5–6 for t6 h, 4–5 for t24 h, 8–10 for t48 h; ±SEM). **b** Properdin-deficient mice have greater bacteremia than wildtype mice at 48 h (*n* = 10 each group, ±SEM)

An intact complement system is necessary in the immune response to encapsulated microorganisms [27]. To investigate the response of properdin-deficient mice to capsular polysaccharides in comparison with wildtype mice, mice were immunised i.p. with Pneumovax, a 23-valent pneumococcal polysaccharide vaccine. Natural antipolysaccharide IgM antibodies were detectable before vaccination. At the end of the four-week experimental period, both genotypes had raised an IgM antiserotype 2 capsular polysaccharide response (Fig. 1a). To determine whether they were protective, immunised mice were challenged with serotype 2 *S. pneumoniae* by i.n. inoculation, which normally proceeds from bronchopneumonia to sepsis within 24–48 h. While both genotypes immunised with Pneumovax were protected in the challenge infection (Fig. 1b), localising pneumococci to alveolar macrophages (Fig. 1c), the survival of the non-immunised mice infected in parallel was significantly different (Fig. 1b): while wildtype mice succumbed rapidly, with 2/5 alive at the experimental endpoint of 7 days, 4/5 properdin-deficient mice remained alive. To confirm this finding, additional groups of naïve mice were infected with an independently prepared stock of passaged *S. pneumoniae*, and again, properdin-deficient mice showed significantly greater survival compared to wildtype mice (8/10 vs 4/10) (Fig. S1A). They also had significantly milder disease at 24 h (Fig. S1B). Individual mice were sampled at various timepoints p.i. and viable pneumococcal counts in lungs and blood determined; while wildtype mice appeared to control bacterial numbers in lungs at 48 h compared to 24 h, as expected for mice of this genetic background, properdin-deficient mice failed to do so (Fig. 2a). In fact, properdin-deficient mice had the highest bacterial count in blood at 48 h (Fig. 2b), from where bacteria may reseed the lungs (personal communication, A. Kadioglu). The differential count of neutrophils in lung homogenates prepared from wildtype and properdin-deficient mice at all timepoints was not significantly different from each other though relative numbers peaked at 24 h. At this timepoint, sublocalisation of neutrophils was investigated histologically (parenchymal vs tethered). There was no difference between the infected genotypes, though parenchymal neutrophils expectedly were elevated compared to controls. The percentage of capillary neutrophils tethered to the endothelium was also comparable between the infected genotypes (Suppl. Table 1). Histological examination of lung sections at this timepoint showed regional leukocytic infiltrations, hyperaemia and thickening of interalveolar spaces, all typical signs of pneumonia. Serum C3 levels and C3 activation fragments in the lung were increased over baseline for both genotypes and were higher for properdin-deficient than for wildtype mice at 48 h (supplementary Table 2), when the viable count was highest in blood and lungs of properdin-deficient mice.

**Fig. 3:**
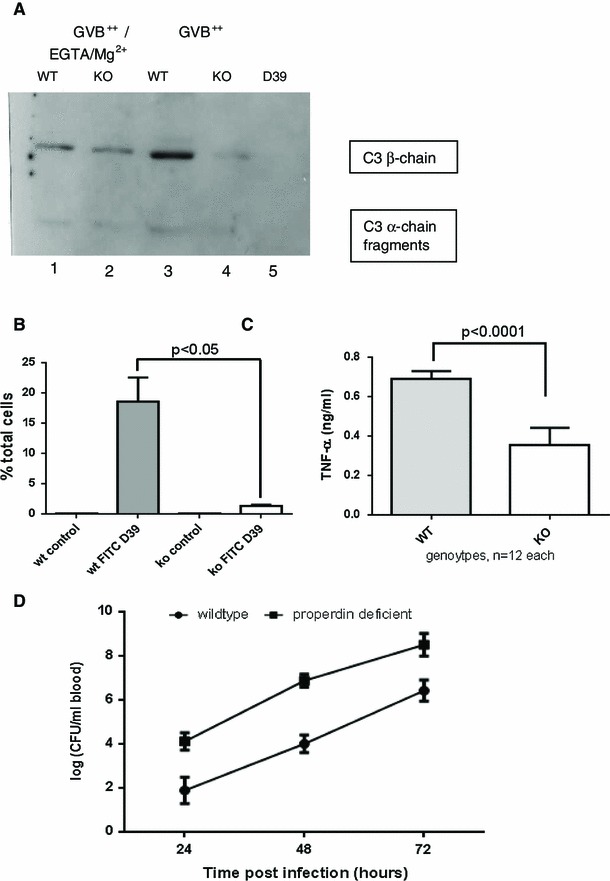
Complement activation, phagocytosis and intravascular survival of *S. pneumoniae* D39. **a** C3 reactivity of separated bacterial pellets after incubation with mouse sera (pool *n* = 6) under conditions favouring the alternative pathway (GVB^++^/EGTA/Mg^2+^) or classical pathway (GVB^++^) activation, 37 °C, 30 min (Western blot); lane 5 shows specificity of rat antimouse C3 antibody (only D39, no serum). **b** Pools of 9 sera-matching each cell genotype were used to opsonise FITC-labelled *S. pneumoniae* before addition to respective mouse splenocytes for 1 h. Flow cytometry was performed, while quenching extracellular signal with trypan blue (representative analysis of *n* = 2). C After 24 h’ infection of splenic macrophages with D39, there is decreased TNF-α production (measured using L929 assay) by splenocytes from properdin-deficient mice in the presence of properdin-deficient sera compared to wildtype cells with wildtype sera. **d** Both genotypes were injected i.v. with 10e6 D39 and bacterial counts (±SEM) determined for each of 3 days from *n* = 5 each genotype (*p* < 0.05)

Previously, activation of the classical pathway of complement was shown to be important in murine survival of *S. pneumoniae* [10]. Wildtype and properdin-deficient mice showed a significant decrease in circulating IgM levels as early as 24 h (Table 1), likely to be due to binding to *S. pneumoniae* and sequestration from the fluid phase. C4c, a product of complement activation, was significantly increased in the sera from infected wildtype, but not in the sera from properdin-deficient mice compared to their respective, comparable, baselines (Table 1), consistent with greater complement activity in properdin-sufficient wildtype mice. In keeping with this, association of C3 to *S. pneumoniae* in vitro via the classical pathway was significantly higher after incubation with sera from wildtype than from properdin-deficient mice (Fig. 3a). To compare opsonophagocytosis in the two genotypes, splenocytes were isolated from wildtype and properdin-deficient mice and incubated for 1 h with *S.pneumoniae* previously incubated with a pool of sera from either wildtype or properdin-deficient mice (Fig. 3b, c). *S. pneumoniae* opsonised with serum from wildtype associated significantly more with splenocytes prepared from wildtype mice compared to the matched properdin-deficient experimental sample. As a consequence, splenocytes from wildtype mice released significantly more TNF-α compared to those from properdin-deficient mice after infection with *S. pneumoniae*. During intravenous infection with *S. pneumoniae*, the rate of bacterial growth from 24 to 72 h was the same in the two genotypes, but the bacterial burden was significantly higher in properdin-deficient mice (Fig. 3d).

**Table 1:** Serum IgM and C4c levels in sera of properdin-deficient and wildtype mice (uninfected and infected with *S. pneumoniae*)

	*T*_0_	*T*_24H_	*T*_48H_
*Serum IgM levels (ng/μl)* ± SD, *p < *0.05 (compared to 0H)*			
Wildtype	88.1 ± 42.4 (*N* = 9)	42.2 ± 28* (*N* = 5)	46.4 ± 4.2* (*N* = 4)
Properdin-deficient	84.1 ± 47.9 (*N* = 10)	45 ± 18.9* (*N* = 5)	52.8 ± 8.1* (*N* = 5)
*Serum C4c levels (semiquantitative, OD*_*405*_ ± SD), *p < *0.05 (compared to 0H)*			
Wildtype	1.6 ± 0.2 (*N* = 3)	1.9 ± 0.5 (*N* = 5)	2.4 ± 0.2* (*N* = 4)
Properdin-deficient	1.5 ± 0.1 (*N* = 2)	1.5 ± 0.6 (*N* = 5)	1.8 ± 0.4 (*N* = 5)

Taken together, the experiments thus far revealed that in the presence of the alternative pathway amplification loop, anticapsular antibodies activate the classical pathway of complement and enhance phagocytosis and proinflammatory cytokine release, the extent of which was detrimental to the wildtype host, while in the absence of properdin, clinical signs of pneumococcal septicaemia were less severe, even though the viable counts recoverable from blood were higher than in wildtype mice.

Following a report that survival of pneumococcal sepsis was improved in the absence of the inhibitory FcγR2b receptor [29]—and others dealing with the crosstalk between complement and FcγR expression [30, 31]—we investigated FcγR2b expression in properdin-deficient and wildtype mice. The mRNA expression of FcγR2b in spleen was reduced in properdin-deficient mice (Fig. S2A). After in vitro infection of splenocytes with *S. pneumoniae*, however, cells from wildtype mice decrease their expression while properdin-deficient cells show more abundant expression, supporting the pro-inflammatory phenotype in wildtype mice shown in Fig. 3c. The activating receptor FcγR4 (functionally antagonistic to FcγR2b), in contrast, is inversely expressed in wildtype and properdin-deficient spleens (Fig S2B). To unequivocally demonstrate a difference in expression of FcγR2b between wildtype and properdin-deficient mice, splenocytes from age- and sex-matched mice were analysed by flow cytometry for expression of FcγR2b. Because the available monoclonal antibodies recognise both FcγR2b and FcγR3, analysis was restricted to B lymphocytes, which only express the inhibitory FcγR2b receptor. Again, properdin-deficient mice had reduced expression of FcγR2b (Fig S2C).

### Properdin shapes the dendritic cell phenotype and contributes significantly to host survival of septicaemia in *Listeria monocytogenes* infection

Because infection with *S. pneumoniae* unexpectedly revealed a cellular phenotype of properdin deficiency (low FcγR2b expression, decreased phagocytosis), mice were next analysed in in vitro and in vivo models using an intracellular pathogen, *L. monocytogenes*. As for *S. pneumoniae*, mouse serum was not bactericidal for *L. monocytogenes* (data not shown), which is consistent with the literature [11, 32].

**Fig. 4:**
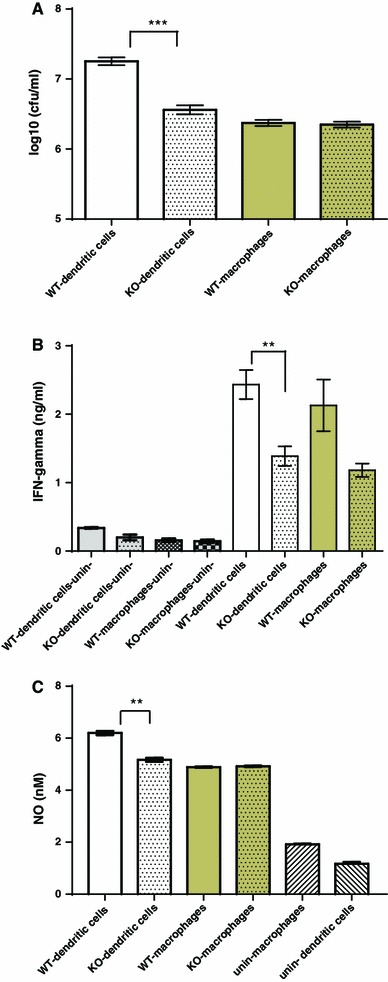
Bone marrow-derived macrophages and dendritic cells infected with *L. monocytogenes*: cellular responses. **a** Intracellular viable counts were determined 24 h p.i., MOI 0.2, after treatment of cells with 10 µg/ml gentamicin (dendritic cell population from wildtype compared to properdin-deficient mice: *p* < 0.005). **b** IFN-γ released into the supernatant of infected cells (24 h) was measured by ELISA (dendritic cell population from wildtype compared to properdin-deficient mice: *p* < 0.01). **c** Nitric oxide release of infected cells (24 h) and controls, determined by the Griess reaction (dendritic cell population from wildtype compared to properdin-deficient mice: *p* < 0.01). Representative of two independent experiments set-up in triplicate

**Fig. 5:**
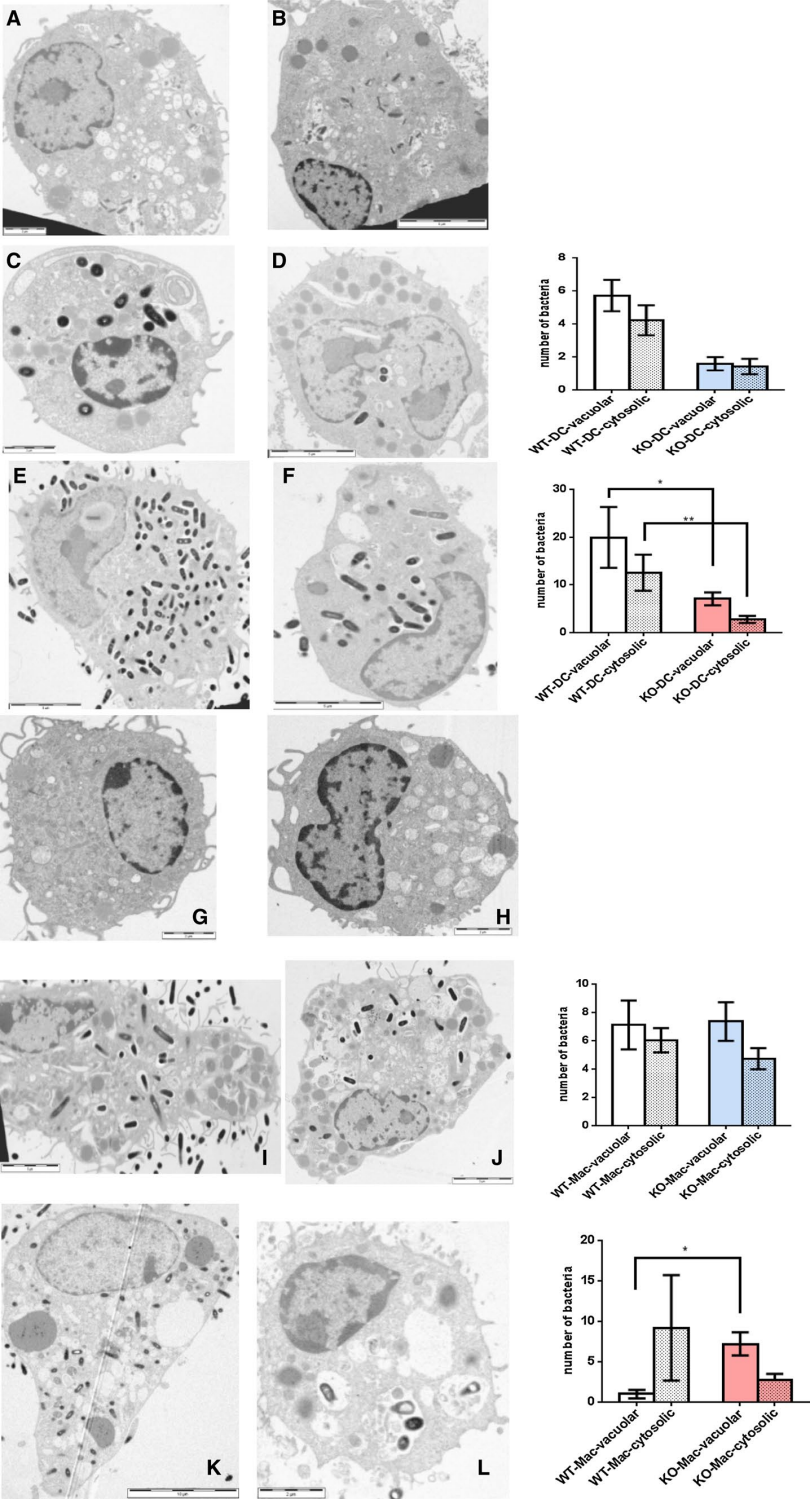
Vacuolar and cytosolic localisation of *L. monocytogenes* in dendritic cells (**a**–**f**) and macrophages (**g**–**l**) from wildtype and properdin-deficient mice (TEM) at 4 h (C,D,I,J) and 24 h (**e**, **f**, **k**, **l**) p.i. Typical electron micrographs are juxtaposed to quantitative analysis of macrophages and dendritic cells from both genotypes (WT-DC *n* = 73; KO-DC *n* = 12 for 4 h; WT-DC *n* = 11; KO-DC *n* = 16 for 24 h, and WT-Mac *n* = 26; KO-Mac *n* = 34 for 4 h; WT-Mac *n* = 5; KO-Mac *n* = 16 for 24 h). Note the overall increase in bacteria at 24 h. *p < 0.05; **p < 0.05 by unpaired *t* test; variables are significantly different (*F* test) for analyses of **c**/**d**, **e**/**f**, **k**/**l**

Bone marrow-derived cells were differentiated in the presence of IL-4 and GM-CSF, yielding an adherent, macrophage-like and non-adherent, dendritic cell-like population [26]. Cells were infected with live *L. monocytogenes.* We found that both cell populations increased their mRNA expression for C3, the integrin CD11b and TLR2 (data not shown). Infected macrophage-like cells from wildtype released marginally more TNF-α than those from properdin-deficient mice when infected with live, not heat killed, Listeria (data not shown). Remarkably, however, the dendritic cell-enriched population from properdin-deficient mice had significantly lower intracellular Listeria compared with those from wildtype mice, while there was no difference in the intracellular burden for the adherent cell population from the two genotypes (Fig. 4a). Release of IFN-γ from infected cells from properdin-deficient mice was lower compared to wildtype mice (Fig. 4b). Nitric oxide release was induced by infection, to the greatest extent in the dendritic cell-like population from wildtype mice (Fig. 4c). Electron microscopy was used to determine the subcellular localisation of *L. monocytogenes*. Consistent with the greater viable counts determined in the dendritic cell-like population from wildtype mice compared with those from properdin-deficient mice, there were more Listeria present in the vacuoles and cytosol of wildtype dendritic cell-like cells (Fig. 5a–f). Macrophage-like cells, however, did not follow this pattern. In contrast, there was a greater number of Listeria in the cytosol at 24 h than in vacuoles of cells from wildtype, not properdin-deficient mice, possibly indicative of lysosomal escape of *L. monocytogenes* (Fig. 5g–l).

**Fig. 6:**
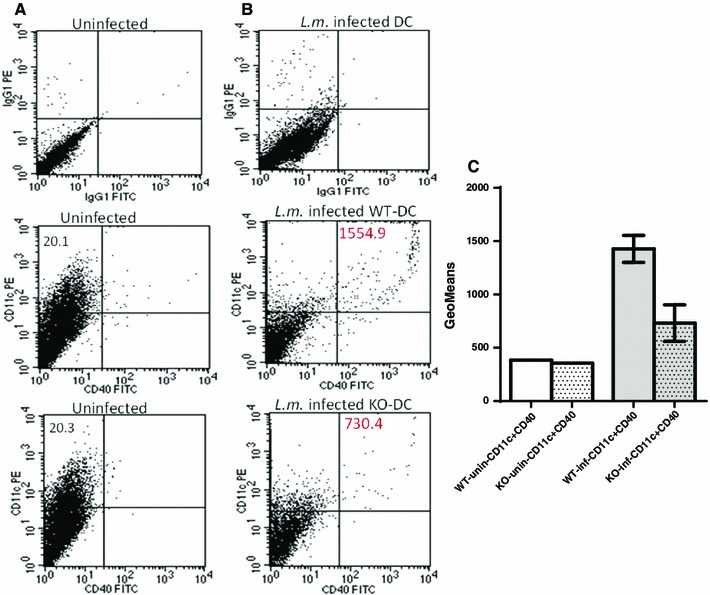
Surface staining for CD40 and CD11c. **a** *Top* isotype controls; *middle* and *bottom* analysis of uninfected non-adherent cell population purified from bone marrow of wildtype and properdin-deficient mice showing 20 % CD11c+. **b** *Top* isotype controls using infected cells to adjust gating; *middle* and *bottom* analysis of infected non-adherent cells from bone marrow of wildtype and properdin-deficient mice. Numbers indicate geometric means of CD11c ^+^ CD40^+^ events. One of three analyses of infected cells is shown. All are summarised in **c**

Listeriolysin was detected in supernatants of infected splenocytes of wildtype and properdin-deficient mice by Western blot (data not shown). Therefore, to preserve the viability of splenocytes for flow cytometry, heat-killed *L. monocytogenes* were used. Compared to the wildtype, the dendritic cell-enriched population of properdin-deficient bone marrow was impaired in the upregulation of CD40, an accessory molecule needed to prolong contact between antigen-presenting and T cells (Fig. 6). Surface expressions of MHCII, CD80 and CD86 were increased after infection, indicating maturation of cells, but were not appreciably different between the genotypes (Fig. S3). A selective impairment of CD40 upregulation in dendritic cells isolated from properdin-deficient mice—contrasting with MHCII, CD80 and CD86—had previously been observed when maturing bone marrow-derived dendritic cells from both genotypes with 1 μg/ml LPS *E.coli* 0111:B4 for 48 h (N. Salehen, PhD thesis).

**Fig. 7:**
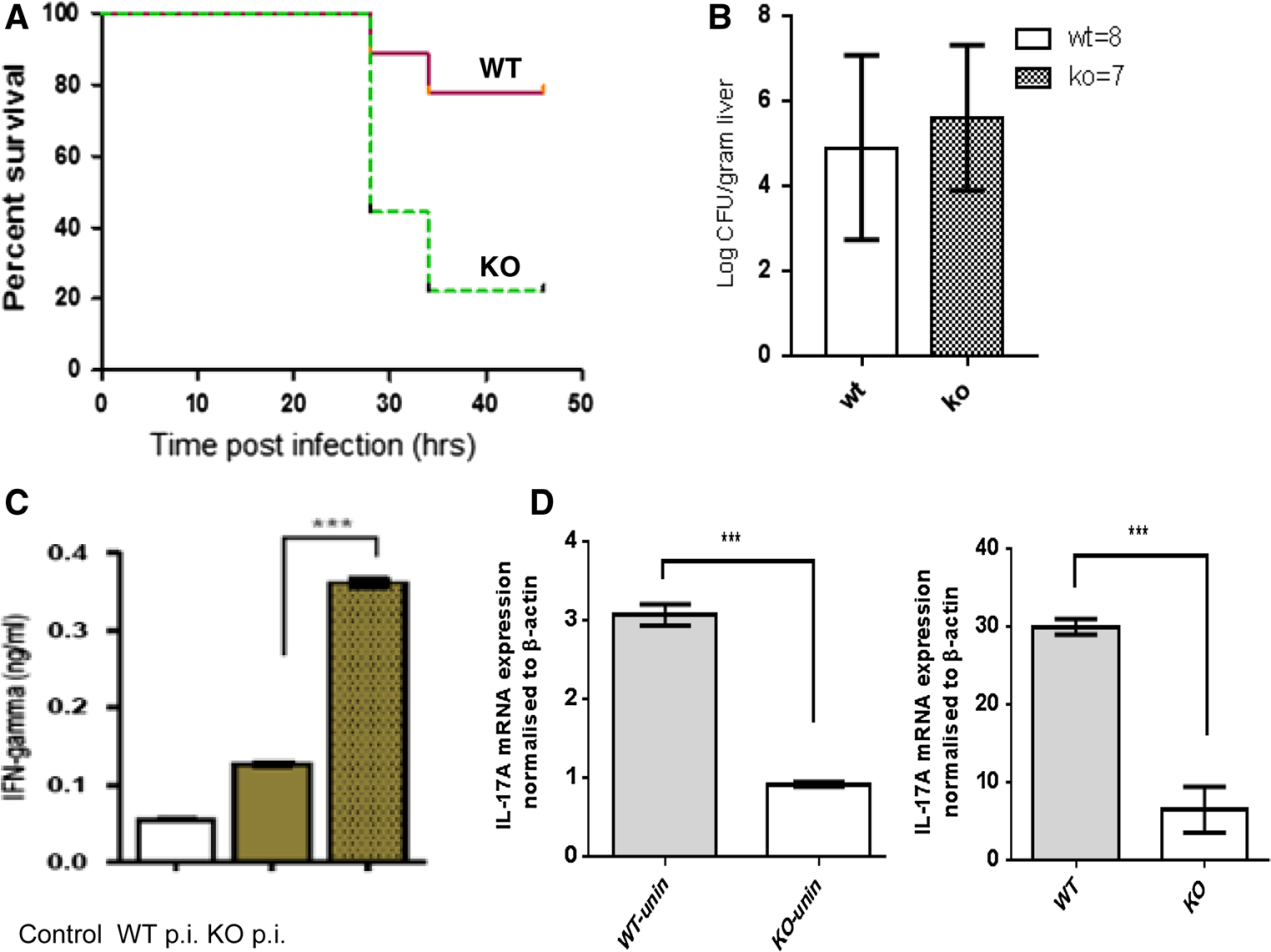
Properdin-deficient and wildtype mice (*n* = 9 each genotype) were injected with passaged *L. monocytogenes* (1 × 10e^6^) and culled at humane endpoint. Survival was plotted against 100 % and was higher in wildtype compared to properdin-deficient mice (*p* < 0.05, **a** viable counts were determined by serial dilution from livers homogenised in PBS; homogenisation in dH_2_O gave the same distribution (**b**). IFNγ was determined by ELISA using sera pooled from mice with identical score of disease severity (lethargic, *n* = 2 each genotype (*p* < 0.0005). **c** IL-17A mRNA expression was quantified from spleens of uninfected mice (*n* = 2, *p* < 0.005) and of mice of equal severity of listeriosis (hunched, piloerect), culled 29 h p.i. (*n* = 1 WT, *n* = 2 KO, *p* < 0.005). Note the increased overall abundance of expression in infected mice (**d**)

An in vivo model of murine listeriosis reproducibly showed that complement properdin contributed significantly to host survival (Fig. 7a) though the numbers of bacteria retrieved from infected livers were not different between the two genotypes (Fig. 7b). After 2 days, 7/9 wildtype mice and only 2/9 properdin-deficient mice were alive. The IFNγ levels in serum were significantly elevated in those mice with worse prognosis, i.e. the *L. monocytogenes* infected properdin-deficient mice (Fig. 7c). Granuloma formation was not impaired in properdin-deficient mice. The titres of antilisterial IgM tested towards immobilised *L. monocytogenes* were comparable in naïve and infected mice (data not shown).

The host normally responds to infection with *L. monocytogenes* with an increase in IL-17 [33], which aids in the polarisation of M1 macrophages [34]. The level of complement activity reportedly affects IL-17 production by T cells and macrophages [35]. When investigating the expression levels in control properdin-deficient and wildtype mice, we found significantly impaired basal expression of IL-17A mRNA in properdin-deficient mice (Fig. 7d). After infection with *L. monocytogenes*, mice of identical severity culled at 29 h were analysed for their splenic IL-17A mRNA expression. Properdin-deficient mice were again reduced in their expression by threefold though the overall abundance of mRNA was higher than unstimulated controls (Fig. 7d). In support of this observation, the relative reduction in inflammatory splenic IL-17A mRNA expression in the absence of properdin was also found when analysing mice infected with a lower dose (5 × 10^5^ *L. monocytogenes*) and survival to 49 h (*n* = 4 WT, *n* = 1 KO) (data not shown).

In summary, this part of the study is the first to show that intact complement is essential in survival of murine listeriosis and in sustaining a cellular response to the intracellular pathogen *L. monocytogenes*. In the absence of properdin, the dendritic cell-like cell population derived from bone marrow had reduced numbers of intracellular Listeria compared to cells from wildtype mice and showed impaired maturation to function as antigen-presenting cells. In vivo, properdin-deficient mice had greater disease severity, consistent with impaired M1-type activation, and worse prognosis than wildtype mice.

## Discussion

Our previous work showing opposing survival outcomes of properdin-deficient mice in response to subacute non-septic shock elicited by LPS or zymosan [7] gave support to the notion that the significance of a role of properdin is tailored to the type of stimulation. Subsequent work using models of zymosan-induced arthritis and collagen antibody-induced arthritis revealed that properdin-deficient mice after intraarticular injection of zymosan showed a Th1 phenotype in regional lymphnodes (resulting in fewer antibodies and more proteoglycan loss in joints) [36], and in the arthritic immune complex disease, less tissue damage was observed, which in part was due to a lack of complement amplification, but also due to a cellular phenotype different from wildtype [37]. Because properdin-deficient mice are not deficient in any of the activation pathways, we surmised that the role of properdin in the outcome from inflammation or infection related to the expression of a predominately humoral or cellular phenotype of the immune response.

Our data indicate that properdin-deficient mice polarise macrophages to an M2 phenotype, which subsequently co-determines host outcome following sepsis. The data add to an accumulating string of evidence that complement activation products shape cellular phenotypes [38–44].

### Reduced mortality of properdin-deficient mice compared to their colony wildtype controls in a streptococcal pneumonia and sepsis model

Previously, we have shown that properdin-deficient mice generated by site-specific targeting [6] were impaired in their alternative pathway activity [6, 7], which led to impaired amplification of the classical pathway of complement activation [36]. Lower levels of C5a in properdin-deficient mice [7, 36] concur with reduced expression of FcγR2b on phagocytic cells, resulting in cells impaired in their ability to phagocytose-encapsulated microorganisms such as *S. pneumoniae* [29].

Mice held in specific pathogen-free environments express natural IgM antibodies reactive with capsular polysaccharides, including those specific for *S. pneumoniae* serotype 2 (Fig. 1a). The anticapsular polysaccharide 2 IgM antibody response after immunisation with the polysaccharide pneumococcal vaccine was comparable between properdin-deficient and wildtype mice. Their total IgM levels were not elevated compared to unvaccinated mice. Mice deficient of C3 or CR2 (the B cell complement receptor) were also able to increase their serotype 2 capsular polysaccharide reactive IgM after immunisation [28], negating concerns that a decrease in C3 fragments would increase the threshold for antibody production [reviewed in [45]]. However, it is worth noting that the immunisations were performed by the i.p. route, stimulating primarily peritoneal B-1-cells [46]. The protective effect of vaccination was tested by i.n. infection with *S. pneumoniae* D39 (serotype 2) and verified for wildtype mice. Properdin-deficient mice, however, reproducibly demonstrated a survival advantage in this model—which was inherent—even though the bacterial load in lungs and systemic seeding into the blood at 48 h post-infection was higher. Their clinical severity scores were significantly better than those obtained for infected wildtype mice.

The increase in serum C3 as an acute phase marker in the inflammatory response showed no difference between the two genotypes. Pulmonary C3 fragments, however, were higher for the infected properdin-deficient group though numbers and location of neutrophils (parenchymal, vascular) were comparable to the infected wildtype group. Given that the bacterial viable count was elevated in lungs of properdin-deficient mice, it is possible that *S. pneumoniae* themselves exerted proteolytic activity against C3, as shown by others [47].

The survival advantage in the absence of properdin is likely to be determined by the impaired ability to phagocytose *S. pneumoniae*, which we have shown using splenic macrophages, leading to reduced activation of macrophages as measured by their reduced release of the pro-inflammatory cytokine TNF-α. These observations are compatible with M2-skewed macrophage activation in the properdin-deficient mice, yielding reduced inflammation and decreased clinical severity. In the septic phase, more IgM is sequestered to the same extent from circulating blood of wildtype and properdin-deficient mice. However, in the absence of the amplification loop of complement, there is no increase of C4c in serum from properdin-deficient compared to wildtype mice at 48 h p.i.

Against these novel findings and given that the response towards zymosan, being rich in protein–carbohydrate complexes, also requires natural antibodies [48], the survival advantage of properdin-deficient mice in our zymosan shock model [7] is likely to have been due to a qualitatively different immune response as outlined above, rather than a beneficially impaired inflammatory ability in the absence of properdin.

### Reduced survival of properdin-deficient mice compared to their colony wildtype controls in a model of murine listeriosis

To understand the role of properdin in cell-mediated responses during infection with *L. monocytogenes*, we investigated phenotypic changes in differentiated bone marrow cells from properdin-deficient and wildtype mice. When infected with the same MOI, the dendritic cell-like population prepared from properdin-deficient mice had reduced numbers of intracellular bacteria compared to that from wildtype mice. As a consequence, these cells were also impaired in their antigenic maturation compared to those from wildtype mice after infection with live *L. monocytogenes* (lower CD40 expression on CD11c^+^ cells). They did not differ in the expression and maturation-induced upregulation of MHCII, CD80 and CD86, thereby expanding from other work, which concluded that C3 was not necessary for maturation of dendritic cells in *L. monocytogenes* infection [39]. CD40, however, was not assessed in these in vitro analyses of dendritic cells from C3 deficient mice [39]. The substantial impairment of CD40 upregulation in properdin-deficient CD11c^+^ cells implies less efficient interactions with cognate T cells. This observation is corroborated by the fact that properdin-deficient mice showed little cytosolic presence of Listeria in their dendritic cell-like population, consistent with impaired cell activation and secretion of IFNγ: cytosolic entry of virulent Listeria is required to increase expression of co-stimulatory molecules and to generate an appropriate T-cell response [49, 50].

M2-skewed myeloid cells express lower CD40, lower IFNγ, TNF-γ and nitrites than M1-polarised cells [51]; this coincides with lower production of IL-17 [52]. Using these parameters, our study gives consistent evidence that cells isolated from properdin-deficient mice retain in culture a spectrum of activity compatible with M2 polarisation.

IFNγ is significantly increased in serum of infected properdin-deficient mice compared to wildtype mice, while both readings were elevated compared to serum from naïve mice. Given that the two genotypes exhibited equal numbers of viable counts in liver homogenates during listeriosis, the increase in IFNγ was likely to be the attempt of properdin-deficient mice to counteract M2-type activity, which is detrimental in listeriosis [53].

**Fig. 8:**
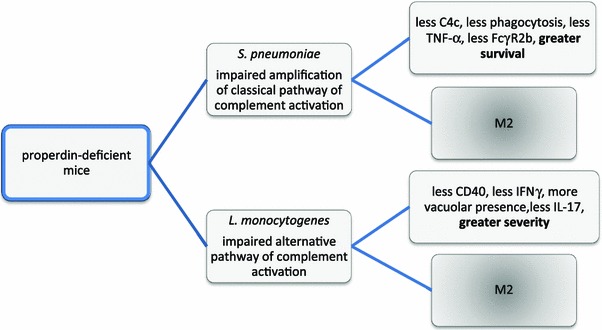
Summary of proposed pathomechanisms of disease in properdin-deficient mice

Taken together, the juxtaposed studies demonstrate that in the attempt to understand the opposing clinical outcomes of the infection models used, a role of properdin deficiency in M2 skewing has been uncovered (Fig. 8). Some determinants of outcome for properdin-deficient mice described for the listeriosis model are likely to have a role in the pneumococcal model via their contribution to M2 skewing, such as IL-17 [54]. Conversely, in wildtype mice, we now conclude that properdin plays a role in maintaining the M1 phenotype favoured by the genetic background of C57Bl/6. Given that there is accumulating literature on a role for complement in cellular immune responses, it follows that the findings presented herein pose relevant considerations in the design of complement targeting biomolecules in general and of properdin blockers in particular.

Finally, though the studies presented herein have advanced the appreciation of the crosstalking role of complement in vivo, ex vivo and in vitro, one relevant contributor to the host response could not be analysed; to profile the virulence of *S. pneumoniae* and *L. monocytogenes* in blood and tissues, an expansion in bacterial growth medium was needed, which provokes another environmental adjustment of the transcriptomes. It is important to recognise that because the pathogens are exposed to selection pressures, which are modulated in the gene-targeted experimental animal, the pathogens are likely to be phenotypically different between the mouse genotypes and therefore are likely to differentially co-determine the infection outcomes in these models. The extent of this contribution is currently under investigation.

## Electronic supplementary material

Below is the link to the electronic supplementary material.

Supplementary material 1 (DOCX 812 kb)

Supplementary material 2 (DOCX 966 kb)
