# Supplementary material for: Septicaemia models using Streptococcus pneumoniae and Listeria monocytogenes: understanding the role of complement properdin
Source: Med Microbiol Immunol. 2014 Apr 12;203(4):257–71. doi: 10.1007/s00430-013-0324-z (PMC4118039; doi:10.1007/s00430-013-0324-z)
Supplement: Supplementary file 1 — Supplementary material 1 (DOCX 812 kb) [file 430_2013_324_MOESM1_ESM.docx]

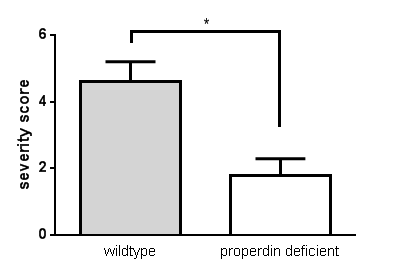
**
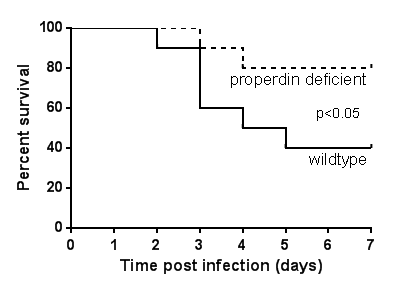
A B**

Figure S1. Properdin-deficient mice have a better outcome from pneumococcal sepsis

A. Properdin-deficient and wildtype mice were inoculated i.n. with 1x 10^6^ CFU WT-passaged *S.* *pneumoniae* D39 (n=10 each) and their survival monitored over seven days; B. Severity of disease signs +SEM 24 hours p.i. (1=normal; 2=hunched, 3=piloerect, 4=lethargic, 5=moribund, 6=dead) for another set of infected mice (n=5 each group) (p<0.05).

**A**

WT spleen

WT splenocytes

unstim (t0)

WT splenocytes +

h.i.D39

KO splenocytes

unstim (t0)

WT splenocytes +

D39

KO splenocytes +

D39

RAW macrophages


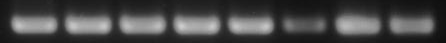

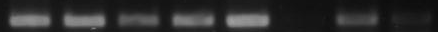


KO spleen

GAPDH

**RT-PCR**

FcγR2b

**
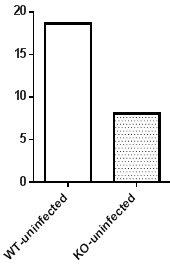

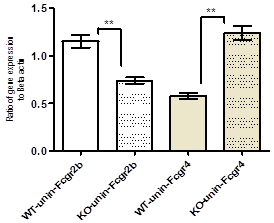
B** **C**

**qPCR**

**MFI CD16/CD32**

**splenic B220^+^**

Figure S2. Properdin-deficient mice are compromised in their expression of FcγR2b.

A. Splenocyte suspensions were prepared from mice and infected overnight with live or heat killed (h.i.) *S. pneumoniae*. RNA expression for FcγR2b (307bp) was analysed semi-quantitatively in comparison with spleen tissues. RAW macrophages (a mouse macrophage cell line) were used as control. B. Quantitative analysis of altered abundance for FcγR2b observed in A using spleens from uninfected mice. FcγR4 mRNA is inversely expressed to FcγR2b, as expected [29]. Representative image of three independent experiments conducted blindly by two individuals (** p<0.01). C. Flow cytometric analysis of FcγgR2b expression on a gated population of B220^+^ cells, expressed as mean fluorescence intensity for the two genotypes analysed in parallel.


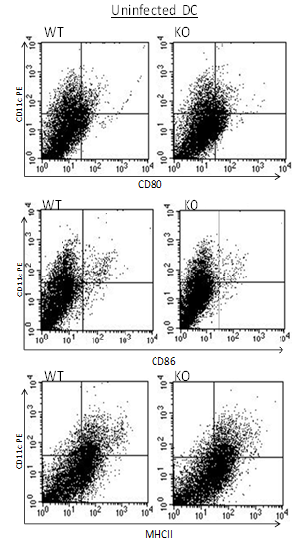

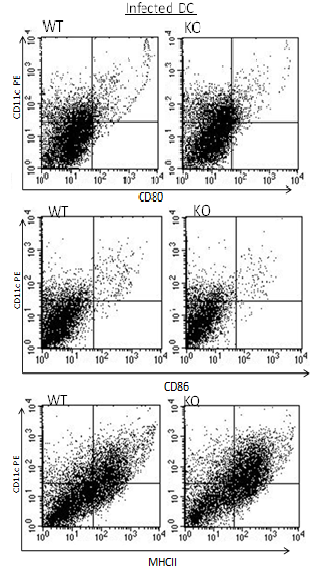

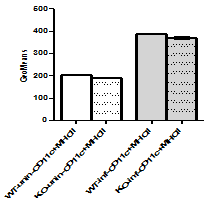

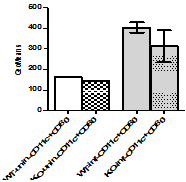

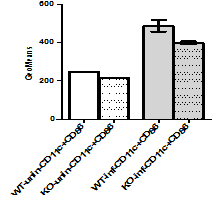

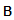

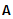


Figure S3. Maturation of dendritic cell populations

Representative dot plots of staining for accessory molecules and MHCII on CD11c^+^ bone marrow derived dendritic cell populations (uninfected, A and infected for 1.5hrs with heat killed *L.monocytogenes*, B), with graphical presentation of geometrical means of surface staining with the specific antibodies for these conditions. 5x10^5^ cells were analysed and graphs are representative of two separate experiments in duplicate.
