# Supplementary material for: Septicaemia models using Streptococcus pneumoniae and Listeria monocytogenes: understanding the role of complement properdin
Source: Med Microbiol Immunol. 2014 Apr 12;203(4):257–71. doi: 10.1007/s00430-013-0324-z (PMC4118039; doi:10.1007/s00430-013-0324-z)
Supplement: Supplementary file 2 — Supplementary material 2 (DOCX 966 kb) [file 430_2013_324_MOESM2_ESM.docx]

Supplementary Table 1

Histological quantification of pulmonary neutrophils in properdin-deficient and wildtype mice

| 24hrs | Parenchymal neutrophils/cm^2^ | Tethered neutrophils (% capillary neutrophils) |
| --- | --- | --- |
| wildtype (n=5) | 8.2 + 2.6 (uninflamed, n=1: 1.6) | 69.6 + 7.3 |
| properdin-def. (n=4) | 6.3 + 1.5 (uninflamed, n=1: 1.5) | 72 + 12.6 |

Exemplary images are shown of pulmonary capillaries (v) filled with erythrocytes; polymorphnuclear granulocytes are indicated (N), tethered to the endothelium, free, and in the interalveolar walls (hematoxylin eosin stain, x100 oil immersion).


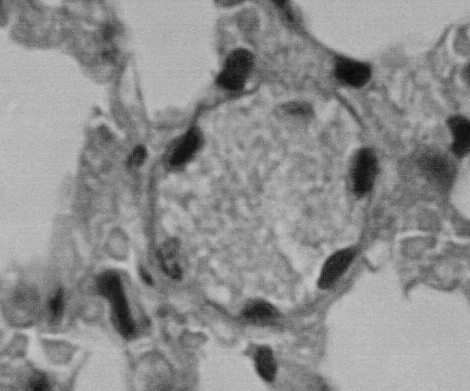

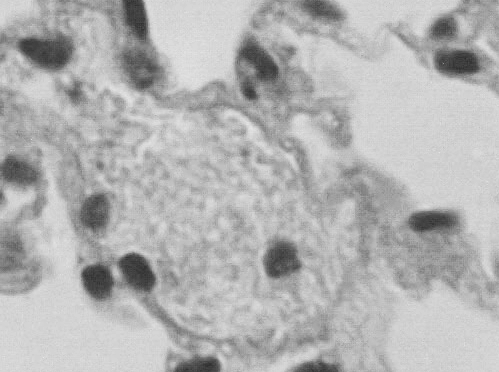


N

N

N

N

N

v

v

Supplementary Table 2:

Serum C3 in properdin-deficient and wildtype mice (uninfected and infected with *S. pneumoniae*)

| ELISA | | 0 hour | 24 hours | 48 hours |
| --- | --- | --- | --- | --- |
| C3 level + SD (mg/ml) | Wild type | 0.58+0.07 (n=2) | 0.84+0.28 (n=5) | 1.06+0.48 (n=4) |
|  | Properdin-deficient | 0.62+0.07 (n=2) | 0.69+0.11 (n=5) | 1.55+0.67 (n=5) |

Analysis of pulmonary C3 activation products in infected mice and controls

| Western blot | | 0 hour | 24 hours | 48 hours |
| --- | --- | --- | --- | --- |
| Pulmonary iC3b/C3dg, arbitrary units of normalised density + SEM | Wild type | 1.07+0.66 (n=2) | 5.74+2.79 (n=5) | 22.82+12.59 (n=4) |
|  | Properdin-deficient | 0.069+0.07 (n=2) | 4.71+3.23 (n=5) | 61.18+18.72 (n=5) |
